# Supplementary material for: 30-year trends in major cardiovascular risk factors in the Czech population, Czech MONICA and Czech post-MONICA, 1985 – 2016/17
Source: PLoS One. 2020 May 11;15(5):e0232845. doi: 10.1371/journal.pone.0232845 (PMC7213700; doi:10.1371/journal.pone.0232845)
Supplement: S1 Data — (PDF) [file pone.0232845.s001.pdf]

Sex

1. Male
2. Female

Education

1. tertiary education: a university degree (any of bachelor/master/higher degrees)
2. tertiary education: a higher professional school/conservatory/undergraduate studies
3. secondary education: gymnasium/secondary technical school/ art academy/conservatory concluded with a final state-standard (maturita) exam
4. basic school education : compulsory school attendance terminated after 9 years regardless of the level achieved
5. secondary vocational school: apprenticeship exams only
6. secondary vocational school: apprenticeship concluded with a final state-standard (maturita) exam
9. not known

Smoking

1. Smoker
2. Non-smoker
3. Ex-smoker
4. Occasional smoker

Awareness of hypertension

HAVE YOU EVER BEEN TOLD BY A PHYSICIAN OR ANY OTHER HEALTHCARE WORKER THAT YOU HAVE DIABETES OR IMPAIRED GLUCOSE TOLERANCE?

1. Yes
2. No
9. Do not know

Medication for hypertension

HAVE YOU USED ANTIHYPERTENSIVE DRUGS WITHIN THE LAST TWO WEEKS?

1. Yes
2. No
3. I do take some drugs but I do not know whether they are antihypertensive agents, or I do not know when I used an antihypertensive agent last.
4. I have never been told I have high blood pressure
9. I do not know

Lipid-lowering drugs

HAVE YOU TAKEN DRUGS LOWERING CHOLESTEROL LEVELS DURING THE PAST TWO WEEKS?

1. Yes
2. No
9. I do not know
