# Supplementary material for: 30-year trends in major cardiovascular risk factors in the Czech population, Czech MONICA and Czech post-MONICA, 1985 – 2016/17
Source: PLoS One. 2020 May 11;15(5):e0232845. doi: 10.1371/journal.pone.0232845 (PMC7213700; doi:10.1371/journal.pone.0232845)
Supplement: Attachment — Submitted filename: Response to Reviewers.docx [file pone.0232845.s003.docx]

Tatsuo Shimosawa, MD, PhD

Academic Editor

PLOS ONE

April 15, 2020

Dear Dr. Shimosawa,

Thank you very much for considering our manuscript for publication. We are very grateful to the reviewers for their valuable comments and suggestions.

Please find below our response to the points raised.

Journal Requirements:

1. Style requirements have been adhered to.

2. The data set with all the individual data from 7 cross sectional surveys have been uploaded as a Supporting information file as well as a file entitled Data_coding_specification.

A link to access the data directly from the hospital server has been established at

<http://www.ftn.cz/data-monica-1117/>

3. The phrase “data not shown” has been deleted as source data have become available.

4. Tables 1 – 5 were included into the main manuscript.

Answers to the Reviewers’ comments”:

Reviewer #1: The comments were mostly related to style and use of English which has been taken into account and an appropriate revision of the text has been done. All the individual typo’s have been corrected. Substantial changes have been introduced in the Discussion section as indicated in the “Revised Manuscript with Track Changes”, pp 11-18.

Reviewer #2: I found this article to be very informative and well written.

Minor comment
The graph shown in Figure 1E gives the impression of upward trend of HDL-C values in females, from 1992 to 2017. However, the temporary downward trend in 2001 (only in age groups 45-65 years) seems to be an unexpected observation. It would be useful to explain it.

We are very grateful for this comment which has lead us to check Table 5. The significance value for HDL-cholesterol for the age group 25-34 years has been, therefore, changed from non-significant to p<0.05 (there was a typing error in the previous Table 5 version).

The text in the **Results** section (pages 10-11, lines….) has been changed as follows:

*Over the same period, there was also a significant but small decline in HDL-cholesterol in males (from 1.35 ± 0.36 to 1.34 ± 0.36 mmol/L; p <0.001) and no significant overall change in females (from 1.57 ± 0.36 to 1.71 ± 0.43 mmol/L; ns). Nevertheless, a significant increase in HDL-cholesterol was observed in the two youngest female age groups. ~~Three-way ANOVA found~~ All the main effects (sex, age groups, year of examination) were found significant (Fig. 1E; three-way ANOVA).*

Appropriate modifications have also been done in the **Discussion** section:

*The decline in total cholesterol in most Western countries was the net effect of an increase in HDL-cholesterol and a decline in non-HDL-cholesterol [33]. ~~However,~~ Contrarily, our 30-year data ~~did not~~ showed just a small increase in HDL-cholesterol in younger females (aged 25-44 years) ~~any HDL-cholesterol changes in females~~ and even a small decrease in HDL-cholesterol in males. This may be due to the fact that healthy lifestyle, including recommended physical activity and refraining from smoking, has been mostly adopted by the younger female population.*

Should there be any further questions, please do not hesitate to contact me.

Yours sincerely,

Renata Cífková

Center for Cardiovascular Prevention Charles University in Prague, First Faculty of Medicine and Thomayer Hospital

Vídeňská 800

140 59 Prague 4

Czech Republic
